# Vein wall thickness and severity of pulmonary involvement due to sars n-cov2 virus infection

**DOI:** 10.1186/s12967-024-04857-w

**Published:** 2024-01-17

**Authors:** Gennaro Quarto, Giacomo Benassai, Annamaria Colao, Antonio Cittadini, Paolo Antonio Ascierto, Rosario Pivonello, Andrea Elefante, Marialuisa Bocchino, Alberto Maria Marra, Ivan Gentile, Gianluca Benassai, Andrea Miletti, Francesca Calemma, Ermenegildo Furino, the members of FACTA study group, Cristina Angelis, Davide Menafra, Francesco Garifalos, Giovanni Domenico De Palma, Maria Carmela Annunziata, Maria Teresa Cantelli, Paola Nappa, Marina Vastarella, Chiara Simeoli, Michele Castoro, Nunzia Verde, Agnese Giaccone, Laura Reynaud, Antonio Riccardo Buonomo, Biagio Pinchera, Chiara Graziadio, Emanuele Filice, Roberta Modica, Rosa Pirchio, Federica Giardino, Roberta D’Assante, Ivo Iavicoli, Luca Fontana, Veruscka Leso, Caterina Nocera, Guido Iaccarino, Antonio Bianco, Eugenio Vaia, Fiore Manganelli, Lucia Ruggiero, Dario Zoppi, Fabio Tortora, Sirio Cocozza, Mario Tortora, Giovanna Muscogiuri, Luigi Barrea, Claudia Vetrani, Alessandro Sanduzzi Zamparelli, Anna Buonocore, Lorena Gallotti, Mauro Mormile, Andrea Bartolomeis, Felice Iasevoli, Annarita Barone, Simone Maurea, Arnaldo Stanzione, Martina Caruso, Claudia Bombace, Maria Triassi, Maddalena Illario, Lorenzo Mercurio, Vincenzo de Luca

**Affiliations:** 1 Department of Clinical Medicine and Surgery, Federico II University, Via Sergio Pansini 5, 80131 Naples, Italy; 2 Department of Translational Medicine, Federico II University, Via Sergio Pansini 5, 80131 Naples, Italy; 3 Melanoma and Cancer Immunotherapy and Developmental Unit, National Cancer Institute IRCCS Fondazione Pascale, Via Mariano Semmola 53, 80131 Naples, Italy; 4 Department of Advanced Biomedical Sciences, Federico II University, Via Sergio Pansini 5, 80131 Naples, Italy; 5 Department of Clinical Medicine and Surgery Section of Respiratory Diseases, Federico II University, Via Sergio Pansini 5, 80131 Naples, Italy; 6 Department for Integrated Activities of Emergency-Admission, Critical Areas and Trauma, Umberto I University Polyclinic – Sapienza University, Viale del Policlinico 155, 00186 Rome, Italy

**Keywords:** COVID-19, Vein wall thickness, ARDS, Chronic venous insufficiency, Chronic venous disease

## Abstract

**Background:**

An observational study involving patients recovered from COVID-19 was conducted in order to evaluate the presence/absence of vein wall thickness increasing, according to the severity of pulmonary involvement quantified with a CT-scoring system.

**Methods:**

The venous wall thickness (VWT) of 31 patients (23 males and 8 females) with COVID 19 previously admitted to Federico II University Hospital of Naples was evaluated through ultrasound measurement of the common femoral Vein 1 cm proximal to the saphenous-femoral junction and the popliteal Vein 1 cm distal to the confluence of gemellary veins. Measurements were taken with an automated tool to avoid human error. All patients were evaluated in the supine position. Patients were then stratified into two groups, VWT > 1 mm and VWT < 1 mm. Lung damage was assessed through thoracic High Resolution Computer Tomography and subsequently quantified using the scoring system set out by Chung et al. CEAP-C class was calculated for all patients.

**Results:**

The mean value of COVID score in VWT > 1 mm group was 7.4 (S.D. 4.83), whilst the mean value of the COVID score in the VWT < 1 mm group was 3.82 (S.D 3.34). These findings were determined to be statistically significant in a two-tie Student-T test. The linear regression test between VWT and Covid score values demonstrated a direct relationship between the two variables.

**Conclusion:**

These results demonstrate a link between two different aspects of the pathological effects on the vessels during a SARS-COV 2 infection. As such a common *primum movens* can be hypothesized in both micro-thrombotic and inflammatory processes relating to COVID 19.

## Introduction

The novel coronavirus SARS-COV-2 outbreak started in Wuhan, China in 2019. As per the latest WHO report, over 200 countries were involved, with more than 146 million confirmed cases and over 3.1 million deaths globally [1].

The virus originates from a bat coronavirus and was transmitted to humans through a zoonotic transfer [2]. As reported in the Lancet, male to female ratio was found to be 50% to 20% [3] and male mortality was 2.4-fold higher than female (70.3 vs 29.7%, P = 0.016) [4].

The clinical manifestations of COVID 19 ranged from asymptomatic to life-threatening. Common symptoms included: fever, sore throat, tiredness, but also vomiting, myalgia and diarrhea [5]. Moreover, liver injury [6], kidney involvement through TMPRSS gene, ischemic and hemorrhagic stroke [7], conjunctival infection [8] were reported.

Microvascular leaks, inflammation, pro-coagulative state and organic ischemia suggest a crucial role of endothelial dysfunction [9]. In fact, the majority of SARS COV 2 infection has been observed in ACE-2 overexpressed cells and CD68- or CD169-overexpressed macrophages [10]. Residues from 318 to 510 in the S1 region are enough for high-affinity binding to peptidase domain of ACE 2 [11]. The wide distribution of ACE2 receptors in arterial and venous endothelial cells might explain various pathophysiologic alterations involving endothelium, resulting in the severity of Covid-19 with different complications, such as the increase of thickness of the vein wall.

During an acute COVID-19 infection, the overactivity of angiotensin II leads to an increase in pro-inflammatory cytokines like IL6, TNF alfa, matrix metallo-proteinases-2, and ROS [12]. Nitric oxide release and prostacyclin reduction exert pro-thrombotic effects and cause oxidative stress resulting in endothelial dysfunction [13].

Endothelial dysfunction leads to an increase in vessel thickness as a result of the immune response [14], inflammatory mediators [15], and ROS [16]. A meta-analysis of 21 studies that includes nearly 2000 patients with Covid 19, reported a prevalence of venous thromboembolism of 37,9% [17].

The aim of this study is to demonstrate if Covid-19 could lead to an increase in VWT as a result of the inflammatory response even in patients without venous thromboembolism and other risk factors.

## Methods

A retrospective study was carried out through analysis of the COVID 19 clinical records of the Federico II University Hospital in Naples. Formal consent to access clinical records was given by the chiefs of Clinical Medicine and Surgery, Translational Medicine and Advanced Biomedical Sciences Departments. A total of 30 patients affected by COVID-19 from March 2020 to October 2021 were considered. 28 patients had acute COVID-19 related symptoms and 24 of those were hospitalized. 3 patients reported serum homocysteine > 15 µM/L, but all were included in CEAP C classes 1 and 2.

Risk factors, such as age at the time of diagnosis, homocysteine levels and venous thromboembolism, were considered.

Lung injury due to SARS n-COV2 was evaluated on the basis of chest High Resolution CT scan images (SIEMENS™ Somatom Drive Dual Source CT ™), using a scoring system described by Chung et al.

In this study, we selected 25 patients, 16 males and 9 females with mean age 52 ± 10.58. 5 patients were not enrolled, because CT images taken during hospitalization were not available.

Initially 200 patients were selected. However, those who already had important comorbidities (Cardiovascular, respiratory, renal, coagulation disorders—such as thrombophilia—and severe chronic venous disease i.e. CEAP-C > 2).) prior to Covid, were excluded from the study. Patients who had previous thromboembolic events and/or Deep Vein Thrombosis (DVT) were not excluded.

No distinction was made according to sex, age and ethnicity.

The inclusion criteria included: long hospitalization, severe respiratory symptoms (dyspnea, cough, fever), cardiovascular and renal complications, need for intensive care.

During hospitalization all the patients underwent blood chemistry tests for the dosage of homocysteine (MAK 34 ™ Sigma Aldrich ™).

After a negative test for SARS n-COV2 infection (ABBOTT MOLECULAR INC™ Alinity™ m SARS-COV-2 amplification reagent kit), patients were contacted for a vascular outpatient clinic appointment which included a lower limb Duplex US. During the Duplex US, we performed a CUS test (compressive US test) and measured venous wall thickness in the supine position.

Duplex US evaluation was performed with 5–18 mHz linear probe (Hitachi™ L64™ on Hitachi™ Arietta 850™) with auto-IMT measurement tool to avoid any human error. Two standardized points of wall thickness measurement were adopted: Common Femoral Vein 1 cm proximal to the saphenous-femoral junction and Popliteal Vein 1 cm distal to the confluence of gemellary veins (where the small saphenous vein opens into the popliteal vein), in major axis projection. Despite the fact that Intima-media thickness (IMT) is a measurement reserved for analysis of the arteries, the automatic detection features of the utilized US device was reliably able to measure the VWT.

We also evaluated the morphology of the thickened segments. Venous segments with increased wall thickness did not show intra-luminal thrombosis. The transition from normal thickness to segments with augmented wall thickness occured gradually, without detachment.

Thickened venous segments appear homogeneously linear in the superficial aspect.

VWT increase is an important risk factor for a thrombo-embolic event, a complication which only 2 patients suffered.

3 patients had homocysteine levels > 15 µM/l, but this risk factor does not directly relate to VWT > 1 mm. 28 patients had acute COVID-19 related symptoms such as ARDS, cough, dyspnea, respiratory failure and 24 of them were admitted to the intensive care unit.

## Results

Patients were divided into two groups (VWT > 1 mm and VWT < 1 mm), and Covid score according to Chung et al. was calculated. SD related to minimal VWT 0,550 and maximal VWT 1,270 was 0,186.

Analysis of variance (VWT Max in mm), parameters of models, equation of model and normalized coefficients were studied. A two-tie T student test with an alpha value of p < 0.05 was performed, to investigate if homocysteine could be involved in VWT increase among the two groups. P value resulted 0,123 (see Tables 1–2).

**Table 1:** VWT-homocysteine: descriptive statistics

Group	OBS	OBS with missing data	MIN	MAX	Average	DEV st
IMT > 1 mm hcy	12	0	5600	48,200	14,633	12,173
IMT < 1 mm hcy	13	0	6000	13,000	9162	1946

**Table 2:** VWT-homocysteine: Student T test

Difference	5472
t (Observed)	1602
|t| (Critical value)	2069
Degrees of Freedom	23
p-value (two-tie)	0123
alfa	005

**Fig. 1:**
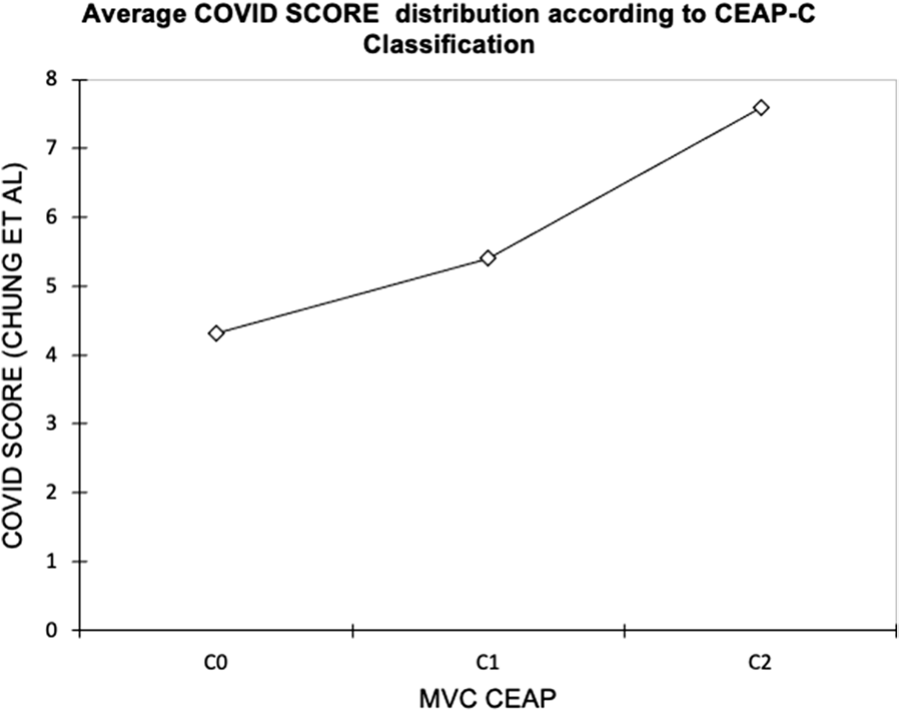
Distribution of COVID SCORE according to the gravity of Chronic Venous Disease (CEAP-C class)

The presence of a correlation between the COVID score with age, gender, degree of impairment of chronic venous disease CEAP-C, and, above all, VWT was evaluated (Fig.1). Linear regressions between COVID score and age and gender did not show a correlation.

**Fig. 2:**
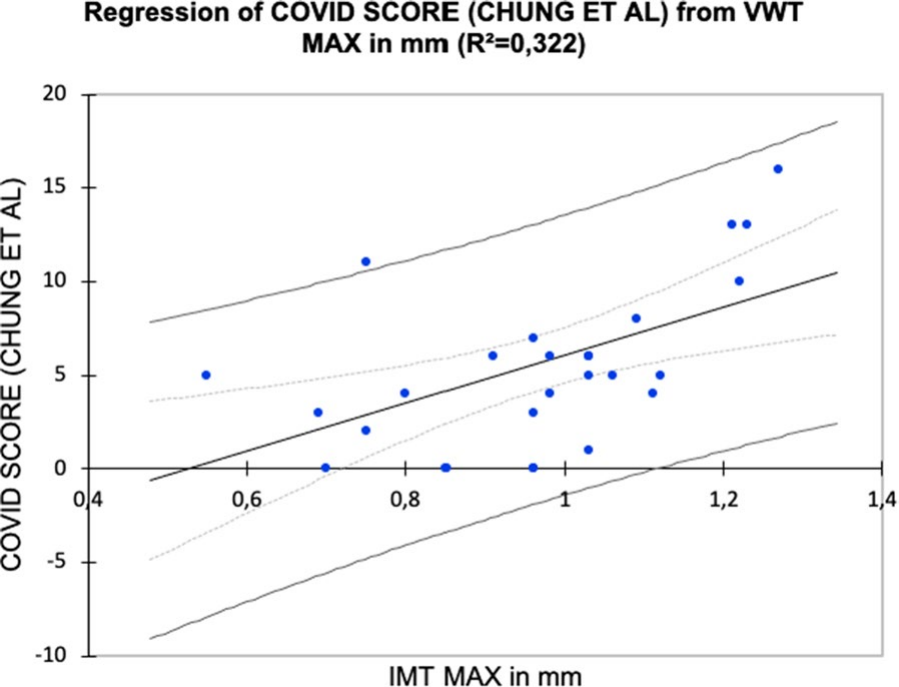
Regression of COVID SCORE (according to Chung et al) from Vein Wall Thickness (expressed in mm)

The ANOVA variance test did not show a link between the COVID score and CEAP-C grading of CVI with an R² value of 0.059, but it is clear and evident that mean COVID score values in patients stratified according to the CEAP grade, assumes a linear trend (Fig. 2 and Tables 3, 4 and 5).

**Table 3:** VWT-CEAP descriptive statistics

Var	CEAP-C	Freq	%
MVC CEAP	C0	3	12,000
	C1	17	68,000
	C2	5	20,000

**Table 4:** VWT-CEAP Matrix of Correlation

	CVD CEAP-C0	CVD CEAP-C1	CVD CEAP-C2	COVID SCORE (CHUNG ET AL)
MVC CEAP-C0	1,000	− 0,538	– 0,185	− 0,124
MVC CEAP-C1	− 0,538	**1**	− 0,729	− 0,109
MVC CEAP-C2	− 0,185	− 0,729	**1**	0,228
COVID SCORE (CHUNG ET AL)	-0,124	− 0,109	0,228	**1**

**Table 5:** VWT-CEAP regression

Obs	25
Sum of weights	25
Degrees of freedom	22
R^2^	0,059
R^2^ corrected	− 0,027
MSE	18,181
RMEQ	4264
MAPE	60,542
DW	2082
Cp	3000
AIC	75,314
SBC	78,970
PC	1198

Linear regression between COVID score and VWT is even more interesting; although not strong (R² = 0.322), it establishes a direct relationship, which is confirmed by a two-tailed T-Test with alpha < 0.05%, with a value of 0.031, with a significant difference between the mean values of COVID score in the groups VWT < 1 mm and VWT > 1 mm, showing higher scoring in the latter group (Tables 6, 7 and Fig. 2).

**Table 6:** VWT-Covid score regression

Obs	25
Sum of weights	25
Degrees of Freedom	23
R^2^	0,322
R^2^ corrected	0,292
MSE	12,533
RMEQ	3540
MAPE	60,016
DW	1955
Cp	2000
AIC	65,125
SBC	67,563
PC	0,796

**Table 7:** VWT-Covid score descriptive statistics

Var	Obse	Obs with missing data	Obs without missing data	Min	Max	Avg	Standard deviation
COVID SCORE (CHUNG ET AL)	25	0	25	0,000	16,000	5720	4208
VWT MAX in mm	25	0	25	0,550	1270	0971	0186

## Discussion

The data resulting from our analysis demonstrate that there is a direct correlation, even if it is not strong, between the grade of severity of the inflammatory involvement of the interstitium and the VWT in patients recovered from COVID-19. This is in line with the susceptibility of the vessel endothelium to the inflammatory insult caused by SARS nCOV2 infection regardless of vessel location.

The novelty of our work lies in the fact that we have correlated venous thickening with the degree of pulmonary inflammation in a non-qualitative but quantitative way.

There are no any other researches, to our knowledge and to date, which have correlated lung inflammation, expressed quantitatively by the Churg scoring, and venous thickening regardless of the occurrence of deep venous thrombosis. Furthermore, in accordance with the above correlation, we investigated about the existence of a link between interstitial pneumonia due to SARS nCoV2 infection and the degree of venous insufficiency and whether one influences the other or vice versa.

Fox et al. [19], reported images showing pulmonary venule thrombosis with immune cell infiltration of thin-walled pulmonary venous circulation. Another study reported subpleural vascular enlargement in 72 of 101 patients with Covid-19 and the authors noted that some lesions were distinct from vascular change in carcinomatosis [20].

Autopsy from patients with severe SARS COV-2 infections found platelet and neutrophil immuno-thrombotic clots even in other organs, which suggest direct embolization [21].

The acute inflammatory response is widely known to cause vascular endothelial injury [22], and the major pathological hallmark of COVID-19 is acute lung injury, resulting in alveolar and microvascular injury [23].

In Quarto et al, 308 patients with chronic venous insufficiency of the lower limbs were investigated in order to evaluate if the fibrotic parietal thickening (> 1 mm) was a reliable parameter of a previous asymptomatic deep vein thrombosis [24]. In this work the hypothesis was that the thickness of the vein wall could be an indirect sign of a previous asymptomatic partial thrombosis of the vessel. In Forauer et al. [25], the inflammatory effects on the endothelium, related to the presence of a permanent venous cannula and the consequent increase in wall thickness were analyzed.

The damage of the venous wall due to fibrosis translates into a vein wall “*meiopragia*” (i.e. weakness) which reduces its elasticity and therefore creates important hemodynamic alterations especially with regards to variations in the intraluminal pressure [26]. The distribution of the Covid score values within the patient stratification according to the CEAP-C class suggests a linear relationship, i.e., the more SARS-related pulmonary compromise increases, the more severity of venous disease increases. This can be read both ways and raises two big questions:

1. Do patients, affected by a pre-existing chronic venous disease, prior to COVID-19 diagnosis, have a coagulative and inflammatory mechanisms that provoke lung damage?

2. Does venous insufficiency, particularly the CEAP-C, detected after COVID-19 infection get worse over time as a result of wall changes, especially in patients who suffered from a major lung damage?

These two questions warrant further investigation regarding the potential utility of venous follow-up of patients affected by COVID 19; moreover, further research is required into a potential common mechanism of endothelial damage in the pulmonary interstitium and lower limbs veins.

The investigated correlations between Chung Covid score and age, sex, homocysteine levels were found to be non-significant.

Above all, the lack of correlation between homocysteine levels and the Chung scoring system, which could have represented an aggravating factor especially in previously diagnosed venous insufficiency (due to the mechanisms of chronic inflammation), eliminates any confounding factor. In fact, according to Rotaru et al, a correlation between homocysteine levels and the Chung COVID Score could have explained, by themselves, the severity of chronic venous disease and the thickening of the venous wall [27]. Hence, increased VWT in patients that suffered major lung damage during acute SARS nCOV2 infection, has implications in terms of progression of chronic venous disease and the need for extensive follow-up and therapeutic interventions in these patients.

## Conclusions

In conclusion, high levels of homocysteine are not related to the damage of pulmonary microcirculation, leading to the hypothesis that other acute inflammatory mechanisms are involved in VWT increase.

The VWT detected in this cohort directly relates to the severity of lung damage from SARS nCOV2.

The distribution trend of the Chung Covid score values suggests a possible link between this trend and the severity of chronic venous disease.

Similarly, although almost the entire study population is vaccinated and/or has been exposed to the virus, therefore, should be immunized and subsequently protected against disease progression. The presence of severe chronic venous disease could be a factor that suggests a greater surveillance of symptoms and their progression, in addition to the already widely codified criteria to identify patients most at risk for severe disease progression.

## Data Availability

The datasets used and/or analyzed during the current study are available from the corresponding author on reasonable request. .
